# Influence of Common Gene Variants on Lipid Levels and Risk of Coronary Heart Disease in Afro-Caribbeans

**DOI:** 10.3390/ijms252011140

**Published:** 2024-10-17

**Authors:** Laurent Larifla, Valerie Bassien-Capsa, Fritz-Line Velayoudom, Vaneva Chingan-Martino, Yaovi Afassinou, Yann Ancedy, Olivier Galantine, Valérie Galantine, Livy Nicolas, Frédérique Martino, Patrick Numeric, Lydia Foucan, Steve E. Humphries

**Affiliations:** 1 Research Team on Cardiometabolic Risk (ECM-RCM), University Hospital of Guadeloupe, 97159 Pointe-à-Pitre, France; valerie.bassiencapsa@chu-guadeloupe.fr (V.B.-C.); fritz-line.velayoudom@univ-antilles.fr (F.-L.V.); vaneva.martino@chu-guadeloupe.fr (V.C.-M.); togbericardo@yahoo.fr (Y.A.); yann.ancedy@chu-guadeloupe.fr (Y.A.); olivier.galantine@chu-guadeloupe.fr (O.G.); valerie.galantine@choisy-cliniques.com (V.G.); nicolas.livy@gmail.com (L.N.); frederic.martino@chu-guadeloupe.fr (F.M.); lfoucan29@yahoo.com (L.F.); 2 Laboratoire de Mathématiques Informatique et Applications (LAMIA), UR 1_1, University of Antilles, 97157 Pointe-à-Pitre, France; 3 Department of Cardiology, University Hospital of Guadeloupe, 97159 Pointe-à-Pitre, France; 4 Department of Rheumatology, University Hospital of Martinique, 97261 Fort-de France, France; patrick.numeric@chu-fortdefrance.fr; 5 Centre for Cardiovascular Genetics, Institute of Cardiovascular Sciences, University College London, London WC1E 6JF, UK; steve.humphries@ucl.ac.uk

**Keywords:** lipids, polygenic risk score, Afro-Caribbean, coronary artery disease

## Abstract

A lower mortality rate from coronary artery disease (CAD) and a more favourable lipid profile have been reported in Afro-Caribbeans compared with people of European ancestry. The aim of this study was to determine whether common lipid variants identified in other populations are associated with lipid levels and CAD in Afro-Caribbeans. We studied 705 Afro-Caribbeans (192 with CAD) who were genotyped for 13 lipid-associated variants. We calculated three polygenic risk scores (PRSs) for elevated LDL (LDL-PRS), decreased HDL (HDL-PRS), and elevated triglycerides (TG-PRS). LDL-PRS, HDL-PRS, and TG-PRS were associated with LDL, HDL, and TG levels, respectively. The LDL-PRS was positively associated with LDL > 2.6 mmol/L and with LDL > 3.0 mmol/L with ORs (odds ratios) of 1.33 (95% confidence interval (CI) = 1.14–1.56) and 1.40 (CI = 1.21–1.62), respectively. The HDL-PRS was associated with a low HDL category (HDL < 1.03 mmol/L) with an OR of 1.3 (CI = 1.04–1.63) and inversely associated with a high HDL category (HDL > 1.55 mmol/L) with an OR of 0.79 (CI = 0.65–0.96). The LDL-PRS was positively associated with CAD after adjustment for age, gender, hypertension, diabetes, and smoking with an OR of 1.27 (CI = 1.06–1.51) but not the HDL-PRS nor the TG-PRS. Results of the present study indicate that common lipid variants are associated with lipid levels and prevalent CAD in Afro-Caribbeans.

## 1. Introduction

Plasma lipids are key factors for the development of cardiovascular disease and particularly coronary artery disease (CAD). There is now a large body of evidence confirming a causal relationship between low-density lipoproteins (LDLs) and atherosclerosis [1]. A low level of high-density lipoprotein (HDL) is also an established risk factor for cardiovascular disease, but medical treatments used to raise the HDL level have failed to reduce atherosclerotic events [2,3]. Similarly, elevated plasma triglycerides (TGs) are associated with an increased risk of cardiovascular disease, but the independent nature of this relationship is still debated [4].

LDL, HDL, and TG blood levels depends on environmental and genetic factors with reported heritability values usually ranging from 30% to 70% [5,6]. Moreover, substantial ethnic differences in lipids concentrations have been reported and may explain the disparity in the risk of CAD between different populations [7,8]. In the last decade, genome-wide association studies have identified numerous single-nucleotide polymorphisms (SNPs) affecting LDL, HDL, and TG levels [9,10,11,12] and have also highlighted differences in effect size and inconsistent replication of genotype–phenotype associations across ethnic groups [13,14,15].

A lower mortality rate from ischemic heart disease has been reported in Afro-Caribbeans (ACs) compared to people of European ancestry (PEA) or Asian Indians [16,17,18], despite the high prevalence of several cardiovascular risk factors such as type 2 diabetes, hypertension, and obesity in the general population. This finding may be due to a more favourable lipid profile (lower small dense LDL, higher HDL, and lower TG) in Afro-Caribbeans [19,20] driven by genetic factors. We have previously reported that the genetic burden for CAD reflected by a 19 SNP polygenic risk score (PRS) was significantly lower in ACs than in PEA, suggesting that genetic factors may explain, in part, the disparity in the risk of CAD between different individuals in the same population as well as between different populations or ethnic groups [21]. However, no genome-wide association study has been performed in ACs, and little data are available on the relevance, in this population, of lipid-altering variants reported in other populations.

In this study, we aimed to investigate if common genetic variants could influence lipid levels and consequently the risk for CAD in ACs.

## 2. Results

### 2.1. Characteristics of the Population (Presented in Table 1)

Obesity (BMI ≥ 30 kg/m^2^) was nearly twice as common in women than in men (33% vs. 17.4%; *p* < 0.001), whereas smoking and regular alcohol consumption were significantly more frequent in men compared to women: 31% vs. 8.7% (*p* < 0.001) and 31.6% vs. 6.1% (*p* < 0.001), respectively.

In participants without CAD that did not receive lipid-lowering agents (N = 473), the mean level of lipid subfractions was 4.92 mmol/L (190 mg/dL) for total cholesterol, 3.08 mmol/L (119 mg/dL) for LDL, 1.36 mmol/L (53 mg/dL) for HDL, and 1.11 mmol/L (98 mg/dL) for triglycerides. There were no significant gender differences in total cholesterol and LDL levels. In contrast, men had a higher triglyceride concentration than women (1.25 vs. 1.00 mmol/L; *p* < 0.001).

### 2.2. Allelic Distribution of Lipid-Altering Variants

For the 13 genotyped SNPs, call rates ranged from 96% to 98%. All SNP were in Hardy–Weinberg equilibrium (*p* > 4 × 10^−3^).

Risk allele frequencies (RAFs) of the SNPs included in the lipid genetic scores and published estimates of PEA are shown in Table 2. The RAFs of the 13 SNPs ranged from 0.08 to 0.94. For eight SNPs, i.e., rs12916 (*HMGCR*), rs1529729 (*SMARCA4*), rs599839 (*SORT1*), rs7412 (*APOE158*), rs301 (*LPL*), rs3916027 (*SLC18A1*/*LPL*), rs1260326 (*GCKR*), and rs17145713 (*BAZ1B*), RAFs were substantially lower in ACs than in PEA. For three SNPs, RAFs were similar (4–5% difference) in the two populations. RAFs were markedly higher in ACs than in PEA for only two SNPs, i.e., rs708272 (*CETP*) and rs17108993 (*RBP4*).

### 2.3. Association of Genetic Risk Scores with Lipid Levels

Distribution of risk allele counts of LDL-PRS, HDL-PRS, and TG-PRS and corresponding lipid levels are shown in Supplementary Material (Figures S1–S3).

We analysed the association of each genetic score with corresponding lipid levels using linear regression models (Table 3) and with risk profile and intervention thresholds using logistic regression models (Table 4).

The LDL-PRS was significantly associated with the LDL concentration and explained 4.6% of the variance, with an increase of 0.144 mmol/L (95% CI = 0.084–0.204; *p* = 2.93 × 10^−6^) or 5.57 mg/dL per additional risk allele. After the incorporation of gender, age, BMI, diabetes, and smoking in the regression model, 11.3% of the variance was captured, and this estimate was similar: +0.154 mmol/L (5.96 mg/dL) per allele (95% CI = 0.095–0.213 mmol/L; *p* = 3.76 × 10^−7^).

The LDL-PRS was significantly associated with an LDL level of >2.6 mmol/L (>100 mg/dL) and with an LDL level of >3.0 mmol/L (116 mg/dL), with sex- and age-adjusted ORs of 1.33 (95% CI = 1.14–1.56; *p* = 3.95 × 10^−4^) and 1.40 (95% CI = 1.21–1.62; *p* = 7 × 10^−6^). After the addition of diabetes, alcohol consumption, and body mass index in the models, these associations remain significant with similar ORs. Participants in the higher tertile of the LDL risk score had an increased risk of crossing the thresholds of LDL > 2.6 mmol/L or LDL > 3.0 mmol/L compared to those in the lower one: OR 2.33 (95% CI = 1.10–4.92; *p* = 0.03) and 2.44 (95% CI = 1.27–4.72; *p* = 0.008), respectively (Table 4).

The HDL-PRS was significantly and inversely associated with (logged) HDL levels with a β effect of −0.021 (95% CI = −0.033, −0.008; *p* = 8.83 × 10^−4^), meaning a 2.1% decrease in mean HDL levels (−0.029 mmol/L (−1.12 mg/dL)) for each additional risk allele. This association remains significant with a similar effect after adjustment for gender, age, BMI, diabetes, and alcohol consumption. The HDL genetic score alone explained 2.4% of the variance of the HDL concentration versus 6.1% after the addition of these factors in the regression model (Table 3).

The HDL score was associated with a low HDL category defined as an HDL of <1.03 mmol/L (40 mg/dL) with an OR of 1.3 (95% CI = 1.04–1.63; *p* = 0.02) and inversely associated with the high HDL category defined by an HDL of >1.55 mmol/L (60 mg/dL) with an OR of 0.79 (95% CI = 0.65–0.96; *p* = 0.02). These associations were similar after adjustment for gender, age, BMI, diabetes, and alcohol consumption. Subjects in the higher tertile of the HDL score distribution had an approximately two-fold increase in the risk of having a low HDL level (OR = 1.99; 95% CI = 1.04–3.79; *p* = 0.04) and inversely a lower probability of having a high cholesterol level (OR = 0.56; 95% CI = 0.32–0.95; *p* = 0.03) (Table 4).

Triglyceride blood levels (logged) were associated with the TG genetic risk score, with each additional risk allele raising the TG level by 1.8% on average, corresponding to a 0.02 mmol/L (1.77 mg/dL) increase in the mean TG level. This association was similar after adjustment for age, gender, diabetes, alcohol consumption, and BMI. The TG score alone explained only 1.6% of the variance of TG while the total variance explained by the model after the addition of the factors mentioned above was 9.4% (Table 3).

The association of the TG-PRS with a TG level of >1.69 mmol/L (150 mg/dL) or with a TG level of >2.26 mmol/L (200 mg/dL) was not significant: OR = 1.14 (95% CI = 0.94–1.37; *p* = 0.20) and OR = 1.18 (95% CI = 0.90–1.55; *p* = 0.24), respectively (Table 4).

### 2.4. Association of Genetic Risk Scores with Coronary Artery Disease

After adjusting for age, gender, hypertension, diabetes, and smoking, the association between genetic risk scores and CAD was significant for the LDL score (OR = 1.27; 95% CI = 1.06–1.51; *p* = 0.009) but not significant for the HDL-PRS (OR = 0.87; 95% CI = 0.71–1.06; *p* = 0.87) nor for the TG-PRS (OR = 1.02; 95% CI = 0.85–1.22; *p* = 0.83).

**Figure 1:**
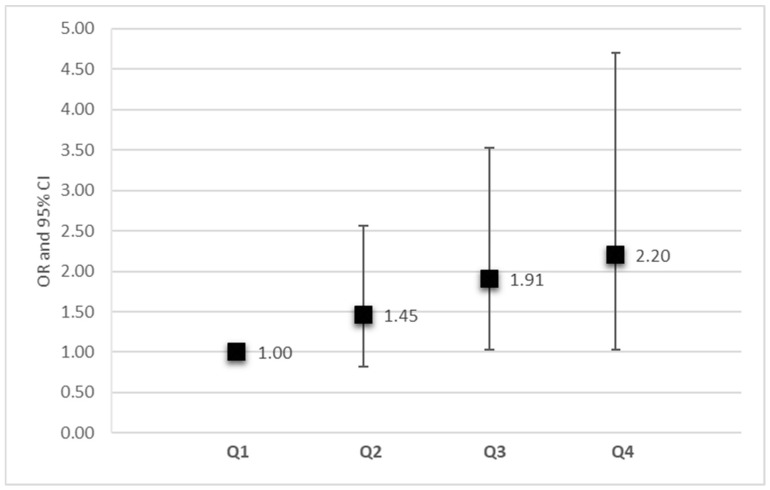
The association between the LDL-PRS divided into quartiles and coronary heart disease. Each quartile of the LDL-PRS, from the first (Q1) to the fourth (Q4), is shown along the x axis. Odds ratios, with the error bars representing the 95% confidence intervals, are plotted on the y axis and were calculated from logistic regression models incorporating age, sex, hypertension, diabetes, and smoking. First quartile is the reference group. *p* values for the second, third, and fourth quartiles are 0.20, 0.04, and 0.04, respectively.

Analysis between subsequent quartiles of the LDL score distribution showed a virtually linear increase in the risk of CAD from the first to the fourth quartile (Figure 1). Participants in the top quartile of the LDL score (11.6% of the sample) had an OR of 2.2 (95% CI = 1.03–4.70; *p* = 0.004) compared to those in the lowest one (36.3% of the sample).

**Figure 2:**
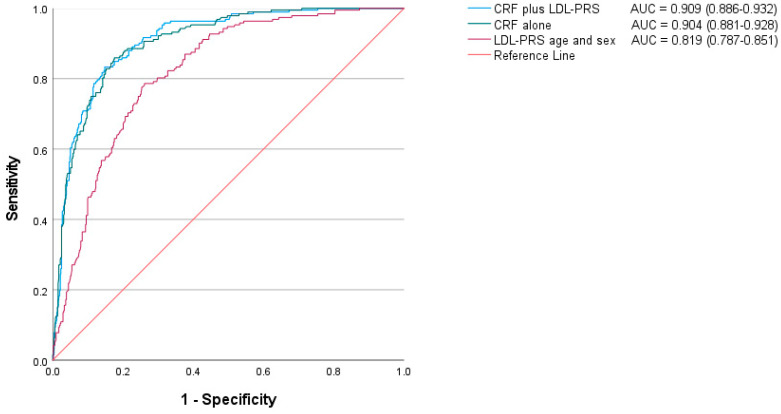
Incremental contribution of the LDL-PRS in coronary artery disease discrimination using receiver operating characteristic area under the curve. Predictions are based on logistic regression models incorporating the LDL-PRS and/or cardiovascular risk factors (age, sex, diabetes, hypertension, and smoking). Areas under the curves (AUCs) are shown on the right side of this figure. CRF: cardiovascular risk factors; PRS: polygenic risk score 3.

The performance of lipid genetic scores in CAD discrimination assessed by receiver operating characteristic area under the curve (AUC) from logistic regression models incorporating cardiovascular risk factors is shown in Figure 2. The LDL score (age- and sex-adjusted) exhibited an AUC of 0.819 (95% CI = 0.787–0.851). Addition of the LDL score in the model incorporating traditional cardiovascular risk factors (age, sex, diabetes, hypertension, and smoking) did not significantly improve the discrimination capacity: AUC 0.909 (95% CI = 0.886–0.932) vs. 0.904 (95% CI = 0.881–0.928); *p* = 0.13.

## 3. Discussion

Several studies have reported a more favourable lipid profile and a lower mortality rate for CAD in ACs compared with PEA [18,19]. In this study, we have demonstrated that three lipid PRSs constructed with common variants that have been previously identified in different populations (mostly European and African American) are associated with the LDL, HDL, and TG blood levels in Afro-Caribbeans. Furthermore, the LDL-PRS was also a strong predictor of CAD in this population. To our best knowledge this is the first report of these findings in Afro-Caribbeans.

In our study, the relatively low mean levels of LDL (3.08 mmol/L) and TGs (1.11 mmol/L) and the relatively high mean level of HDL (1.36 mmol/L) are in line with previous reports showing a favourable lipid profile in people of African ancestry compared to PEA with higher levels of HDL and lower levels of triglycerides, VLDL, and small dense lipoprotein [16,27,28,29,30]. These ethnic differences in lipid profiles have also been observed in children and adolescents [31] and support the hypothesis that polymorphisms in lipid metabolism genes might be involved in these disparities [27].

In this study, for most of the SNPs, the risk allele frequency was much lower in ACs than in PEA, while the opposite was true for only 2 SNPs. Although only a limited number of lipid-altering variants have been tested in our study, these findings suggest that the favourable lipid profile observed in ACs compared to PEA may be related, in part, to differences in common variant distribution.

Moreover, the interethnic differences in lipid profiles may also be related to differences in effect size of the associated variants. Deo et al. analysed a panel of 12 lipid-altering variants discovered in PEA in a cohort of 4464 African Americans from the Jackson Heart Study and reported a lower effect size in this population for all the studied variants [13].

In our study, the LDL-PRS, HDL-PRS, and TG-PRS constructed with 5, 4, and 7 SNPs were all associated with the lipid levels but captured only 4.6%, 2.4%, and 1.2% of the unadjusted variance of LDL, HDL, and TGs, respectively. Although representing a small proportion of the variance of the corresponding lipid level, the contribution of the LDL and HDL polygenic risk scores was superior compared to that of the other traditional risk factors including BMI and age. These proportions are lower but similar to those reported by Murray et al. who analysed a sample of 635 Italian individuals over 65 years of age and found that a LDL-PRS (based on 7 SNPs), a HDL-PRS (based on 9 SNPs), and a TG-PRS (based 11 SNPs) explained 7.1%, 3.4%, and 4.8% of the variance of the corresponding lipid fraction level, respectively [32].

Large observational studies indicate strong associations between the LDL, HDL, and TG levels and the risk of coronary heart disease [33,34].

Genome-wide association study (GWAS) and meta-analysis of candidate gene studies have identified numerous variants in several genes that are associated with both the lipid phenotype and CAD such as *SORT1*, *LPA*, *LPL*, *APOA5*, *APOB*, *APOC3*, *APOE*, *ANGPTL3*, *PCSK9*, *NPC1L1*, and *HMGCR* [35].

Only few studies, mostly in PEA, have demonstrated the combined effect of lipid-altering variants on the risk of CAD using genetic risk scores. A genetic score including nine SNPs associated with higher LDL cholesterol or lower HDL levels was associated with incident cardiovascular disease in 5414 Swedes [9].

Shah et al. have also demonstrated, using a genetic score approach, that common SNPs associated with LDL and TGs contributed to blood lipid variation and cardiovascular risk in two British cohorts of 5059 and 3414 individuals [23]. A similar finding was reported by Murray et al. in an older European cohort (635 Italian aged 65 years and older) [32]. More recently, Shahid et al. compared the effects of *SORT1*, *APOB*, and *APOE* variants on the LDL levels and CAD in 623 Pakistani subjects compared to a British cohort. In this study, although there were significant differences in allele frequency distribution between the two populations, each SNP was associated with the LDL level, and the gene score was associated with CAD in both populations [36].

In our study, we were able to detect a strong association between lipid PRSs, lipid levels, intervention thresholds, and the risk of CAD despite a limited number of subjects in an Afro-Caribbean population presenting a relatively favourable lipid profile and a lower effect size compared to those of PEA. This highlights the interest of the use of genetic markers summarised in genetic scores for the risk stratification of complex diseases such as CAD. However, the addition of the lipid PRS did not significantly improve the prediction of a model that incorporated traditional risk factors for CAD. This could be due to the limited size of our sample and the use of a genetic score composed exclusively of lipid-altering variants that represent only a part of the numerous CAD-associated variants. In this line, Iribarren et al. have demonstrated the clinical utility and the incremental value of the incorporation of a multilocus gene score into the Framingham risk equation for the prediction of CAD in a cohort of more than 51 900 Europeans [37].

### Limitations

Limitations of this study include the relatively small sample size.

Moreover, the number of studied SNPs is not exhaustive since associations with other variants have been identified in GWAS [11]

We used a simple allele count for the lipid PRS calculation as no estimate of the different variants was available in our population, and we cannot disregard the fact that the use of an appropriate weighted PRS would be more accurate to evaluate the combined effects of the different variants on the lipid levels and the risk of CAD.

For the calculation of the LDL cholesterol concentration, we used two different formulas: the Friedewald equation was used when the TG levels were below 3.9 mmol/L (340 mg/dL) and the Planella equation when the TG levels were above that value. However, in patients with high TG levels (>4.52 mmol/L (400 mg/dL)), two other equations, the Martin–Hopkins and Sampson equations, are currently used in the clinical practise and their performance has been more widely evaluated than that of the Planella equation. In our study, subjects with a TG concentration of >3.9 mmol represented only a small proportion (1.8%) of the sample; therefore, it is unlikely that the selective use of these two validated equations may have altered the validity of our results.

The strengths of this study include that it was conducted on a sample of Afro-Caribbean subjects given that the effect of specific genetic variants for the coronary risk assessment can differ across populations and that the related studies have been mostly conducted in populations of European ancestry.

## 4. Materials and Methods

### 4.1. Study Population

We analysed a set of data of 705 Afro-Caribbeans (including 192 with CAD) from a previously described prospective case–control study [38].

Subjects without any cardiovascular disease, including myocardial infarction, acute coronary syndrome, peripheral artery disease, and stroke, were recruited from two units of medicine at the university hospitals of Guadeloupe and Martinique and a medical health centre in Guadeloupe. Guadeloupe and Martinique are two French overseas departments and neighbouring islands located in the Caribbean that share similar socioeconomic status and ethnic distribution with a majority of Afro-Caribbeans.

Patients with CAD (hospitalised for CAD and/or with a documented history of acute coronary syndrome according to the World Health Organization criteria or a coronary stenosis, or a coronary angioplasty, or a coronary artery by-pass surgery) were recruited from the department of cardiology of the University Hospital of Guadeloupe.

The ethnic origin was determined when the patient defined him/herself and his/her two first-degree relatives as Afro-Caribbeans. The study was approved by the regional ethics committee (Sud-Ouest/Outre-Mer III, France, N° 06/0806, 30/07/2008). All patients gave their written informed consent to participate in this study.

### 4.2. Data Collection

Data on age, sex, height, weight, and use of antihypertensive and/or hypolipidemic treatment were collected during the enrolment visit. Body mass index (BMI) was calculated as weight divided by height squared (kg/m^2^). Systolic and diastolic blood pressure readings were assessed using an automated monitor after the subject had rested for at least 5 min. The recorded values were the average of two measurements in each arm.

### 4.3. Biochemical Analyses

The collection of blood samples was conducted within a week (generally less than 24 h) after the formal enrolment. Blood samples were collected after an overnight fast of >10 h. The concentrations of glucose, total cholesterol, HDL, and triglycerides were measured using an automatic multichannel chemical analyser (Cobas Integra 800, Roche Diagnostic, Meylan, France). The low-density lipoprotein (LDL) cholesterol concentration was calculated using the Friedewald formula in subjects with triglyceride levels of <3.9 mmol/L (340 mg/dL). In subjects with triglyceride levels ≥ 3.9 mmol/L, the LDL concentration was calculated using the Planella formula based on apolipoprotein B, given the lake of accuracy of the Friedewald formula at high TG levels.

### 4.4. Genotyping and Lipid Genetic Score Calculation

Genotyping was performed using published TaqMan (Applied Biosciences/Life Technologies, Grand Island, NY, USA) and KASPar (KBioscience, Hertfordshire, UK) technologies.

We selected 13 SNPs that were associated with the LDL, HDL, or TG levels in genome-wide association studies (GWASs) or meta-analysis of candidate gene association studies. For each lipid fraction, only SNPs with a documented association in people of African origin/African American or alternatively in at least two different ethnic groups were selected.

Three PRSs (LDL-PRS, HDL-PRS, and TG-PRS) were calculated for each subject by summing the number of risk alleles at each locus (0: if a risk allele is absent; 1: if the risk allele is a heterozygote; or 2: if the risk allele is a homozygote).

The SNPs used to construct the genetic scores included five SNPs (rs12916 (*HMGCR*), rs1529729 (*SMARCA4*), rs599839 (*SORT1*), rs429358 (*APOE*), and rs7412 (*APOE*)) for LDL, four SNPs (rs301 (*LPL*), rs3916027 (*SLC18A1*/*LPL*), rs17231506 (*CETP*), and rs708272 (*CETP*)) for HDL, and 5 SNPs (rs1260326 (*GCKR*), rs17145713 (*BAZ1B*), rs3916027 (*SLC18A1*/*LPL*), rs328 (*LPL*), and rs17108993 (*RBP4*)) as well as the *APOE* haplotype for TGs.

### 4.5. Definition of Clinical Factors

Obesity was defined as a BMI of ≥30 kg/m^2^. Hypertension was defined as a systolic blood pressure of 140 mmHg or a diastolic blood pressure of 90 mm Hg or the current use of antihypertensive medication.

Participants were considered as current smokers if they were regularly smoking more than one cigarette per day at the time of inclusion; they were considered as having diabetes if they had a history of diabetes or were prescribed hypoglycaemic agents (including insulin); they were considered as having hypercholesterolemia if they had a history of hypercholesterolemia or were prescribed a lipid-lowering therapy; they were considered to exhibit regular alcohol consumption if they drank alcohol on more than three days per week.

### 4.6. Statistics

The data are presented as mean ± standard deviation for continuous variables and as number (percentage) for categorical variables.

Linear regression models were used to test the association of the PRS with the lipid levels. HDL and TG variables, which presented a skewed distribution, were log-transformed prior t I confirm o analysis. Participants on lipid medication and/or with coronary artery disease were excluded from this analysis.

To determine the impact of the PRS on lipid profiles, we selected several cut-off values of LDL, HDL and TGs, defining the different risk categories for CAD and representing intervention thresholds according to the European Society of Cardiology guidelines and the NCEP/ATP III report [39,40]: LDL > 100 mg/dL (2.6 mmol/L), LDL > 116 mg/dL (3.0 mmol/L), HDL < 40 mg/dL (1.03 mmol/L), HDL > 60 mg/dL (1.55 mmol/L), TG > 150 mg/dL (1.69 mmol/L), and TG > 200 mg/dL (2.26 mmol/L).

Using logistic regression models, we calculated the odds ratio (OR) for a given lipid profile. The lipid PRSs were treated as continuous variables. We also divided the lipid PRS distributions into tertiles and compared the participants in the lowest tertile to those in the highest one.

Similarly, we used logistic regression models to assess the association of the lipid PRS with CAD.

For both linear and logistic regressions, we used unadjusted models, age-adjusted and age- and sex-adjusted models, and fully adjusted models including all variables of interest for each PRS. In fully adjusted models, we considered the variables that captured the greatest variance.

To evaluate the predictive value of the lipid PRSs for CAD, we used the receiver operating characteristic curve analysis and calculated the area under the curve (AUC) from logistic models adjusted for age, sex, and cardiovascular risk factors.

IBM SPSS Statistics, version 27 (IBM Corp., Armonk, NY, USA), was used for analysis. All tests were two-sided, and *p* values of <0.05 were considered significant.

## 5. Conclusions

Genetic variants are associated with the lipid levels and the risk of coronary artery disease in Afro-Caribbeans. Further studies are needed to evaluate the clinical utility of lipid genetic risk scores for assessing the need of lipid-lowering medication and the risk of coronary events.

## Figures and Tables

**Table 1:** Characteristics of the study sample.

	All	Males	Females	*p*
**N (%)**	705	348 (49.4)	357 (50.6)	
**Age**	53.71 (14.02)	53.97 (13.13)	53.45 (14.85)	0.62
**BMI (kg/m^2^)**	27.06 (5.46)	25.88 (4.27)	28.20 (6.21)	1.23 × 10^−8^
**Obesity (BMI ≥ 30 kg/m^2^)**	175 (25.3)	59 (17.4)	116 (33.0)	2.10 × 10^−6^
**Hypertension**	286 (40.6)	140 (40.2)	146 (40.9)	0.86
**Diabetes**	122 (17.3)	65 (18.7)	57 (16.0)	0.34
**Smoking**	139 (19.7)	108 (31.0)	31 (8.7)	8.81 × 10^−14^
**Regular alcohol consumption**	125 (18.5)	104 (31.6)	21 (6.1)	2.25 × 10^−18^
**C** **oronary artery disease**	192 (27.2)	120 (34.5)	72 (20.2)	1.97 × 10^−5^
**Use of lipid-lowering agents**	185 (26.2)	103 (29.6)	82 (23.0)	0.045
**N (%)**	473	212 (44.8)	261 (55.2)	
Lipid levels (mmol/L) *				
**Total cholesterol**	4.92 (1.05)	4.88 (1.13)	4.95 (0.99)	0.48
**LDL cholesterol**	3.08 (0.89)	3.02 (0.92)	3.14 (0.88)	0.14
**HDL cholesterol**	1.36 (0.48)	1.32 (0.52)	1.38 (0.45)	0.06
**Triglycerides**	1.11 (0.73)	1.25 (0.84)	1.00 (0.61)	0.001

Qualitative variables are expressed as number (percentage) and quantitative variables as mean (standard deviation). * Data on lipid levels are from participants without CAD and not receiving lipid-lowering agents. HDL and triglycerides were log-transformed for this analysis.

**Table 2:** Risk allele frequencies of the SNPs included in the polygenic risk score calculation for Afro-Caribbeans (study sample) and their published estimates in people of European ancestry.

SNP	Gene/Locus	Chr	Alleles (Risk/Reference)	RAFs in ACs (Study Sample *)	RAFs in PEA (Published Data)	β per Risk Allele in PEA (mmol/L) (Published Data)	*p* Value **	Reference
LDL-raising variants								
rs12916	*HMGCR*	5	C/T	0.24	0.38	0.02	1.40 × 10^−11^	[22]
rs1529729	*SMARCA4*	19	C/T	0.29	0.45	0.09	6.71 × 10^−6^	[23]
rs599839	*SORT1*	1	A/G	0.23	0.77	0.14	6.10 × 10^−33^	[12]
rs429358	*APOE112*	19	C/T	0.24	0.19	0.24	5.82 × 10^−11^	[24]
rs7412	*APOE158*	19	C/T	0.91	0.96	0.49	1.8 × 10^−31^	[24]
HDL-lowering variants								
rs301	*LPL*	8	C/T	0.29	0.75	−0.04	9.25 × 10^−11^	[23]
rs3916027	*SLC18A1*/*LPL*	8	G/A	0.59	0.73	−0.04	5.39 × 10^−10^	[11]
rs17231506	*CETP*	16	C/T	0.73	0.68	−0.07	2.21 × 10^−36^	[23]
rs708272	*CETP*	16	C/T	0.76	0.57	−0.06	1.87 × 10^−32^	[11]
TG-raising variants								
rs1260326	*GCKR*	2	T/C	0.16	0.41	0.10	5.68 × 10^−133^	[25]
rs17145713	*BAZ1B*	7	C/T	0.69	0.80	0.09	5.29 × 10^−10^	[11]
rs3916027	*SLC18A1*/*LPL*	8	G/A	0.59	0.73	0.08	1.04 × 10^−10^	[11]
rs328	*LPL*	8	C/G	0.94	0.89	0.11	8.42 × 10^−10^	[11]
rs17108993	*RBP4*	10	G/T	0.16	0.03	0.14	8.04 × 10^−6^	[11]
rs429358	*APOE112*	19	ε2	0.08	0.07	ε2/ε2 vs. ε3/ε3 mean difference: 0.34 mmol/L (95% CI = 0.18–0.50)		[26]
rs7412	*APOE158*	19						

Beta coefficient represents the change in lipid levels expressed in mmol/L. SNP: single-nucleotide polymorphism; RAF: risk allele frequency; Chr: chromosome; ACs: Afro-Caribbeans; PEA: people of European ancestry. * Risk allele frequency among people (Afro-Caribbeans) without coronary artery disease. ** *p* values for the association between lipid-altering SNPs and associated lipid fraction levels.

**Table 3:** Association of genetic scores and lipid levels in linear regressions models.

Lipid Genetic Score	Lipid Level	Unadjusted			Adjusted		
	(mmol/L)	Per Risk Allele Effect (β Effect (95% CI))	*p* Value	Explained Variability (%)	Per Risk Allele Effect (β Effect (95% CI))	*p* Value	Explained Variability (%)
LDL-PRS	LDL	0.144 (0.084, 0.204)	2.93 × 10^−6^	4.6	0.154 (0.095–0.213)	3.76 × 10^−7^	11.3
HDL-PRS	LogHDL	−0.021 (−0.033, −0.008)	8.83 × 10^−4^	2.4	−0.020 (−0.032, −0.008)	6.6 × 10^−5^	6.1
TG-PRS	LogTG	0.018 (0.002, 0.033)	0.02	1.2	0.018 (0.004, 0.033)	0.015	9.4

Only data from subjects with no coronary artery disease who were not receiving lipid-lowering medication were considered in the analysis (N = 473). The HDL and TG levels were log-transformed. LDL-PRS was adjusted for gender, age, body mass index, diabetes, and smoking. HDL-PRS was adjusted for gender, age, body mass index, diabetes, alcohol consumption. TG-PRS was adjusted for gender, age, body mass index, diabetes, alcohol consumption, and hypertension. PRS: polygenic risk score.

**Table 4:** Association of genetic risk scores with risk profile and intervention threshold.

	PRS (Risk Allele Count) (Age- and Sex-Adjusted)		PRS (Risk Allele Count) (Fully Adjusted *)		Tertiles of PRS (Age- and Sex-Adjusted)		Tertiles of PRS (Fully Adjusted *)	
					Upper tertile vs. lower tertile		Upper tertile vs. lower tertile	
	OR (95% CI)	*p*	OR (95% CI)	*p*	OR (95% CI)	*p*	OR (95% CI)	*p*
**LDL-PRS**								
LDL > 2.6 mmol/L (100 mg/dL)	1.33 (1.14–1.56)	3.95 × 10^−4^	1.35 (1.15–1.59)	3.22 × 10^−4^	2.33 (1.10–4.92)	0.03	2.38 (1.12–5.07)	0.02
LDL > 3.0 mmol/L (116 mg/dL)	1.40 (1.21–1.62)	7 × 10^−6^	1.44 (1.23–1.68)	3.58 × 10^−6^	2.44 (1.27–4.72)	0.01	2.56 (1.31–5.01)	0.006
**HDL-PRS**								
HDL < 1.03 mmol/L (40 mg/dL)	1.30 (1.04–1.63)	0.02	1.29 (1.03–1.61)	0.03	1.99 (1.04–3.79)	0.04	2.02 (1.04–3.92)	0.04
HDL > 1.55 mmol/L (60 mg/dL)	0.79 (0.65–0.96)	0.02	0.79 (0.64–0.97)	0.02	0.56 (0.32–0.95)	0.03	0.56 (0.32–0.98)	0.04
**TG-PRS**								
TG > 1.69 mmol/L (150 mg/dL)	1.14 (0.94–1.37)	0.20	1.14 (0.94–1.38)	0.18	-------------------		--------------	
TG > 2.26 mmol/L (200 mg/dL)	1.18 (0.90–1.55)	0.24	1.18 (0.90–1.55)	0.24	------------------		--------------	

* Age, gender, diabetes, smoking, and body mass index adjusted for LDL-PRS; age, gender, diabetes, alcohol consumption, and body mass index adjusted for HDL-PRS; age, gender, diabetes, alcohol consumption, body mass index, and hypertension adjusted for TG-PRS; PRS: polygenic risk score.

## Data Availability

The original contributions presented in the study are included in the article/Supplementary Material, and further inquiries can be directed to the corresponding author.

## Supplementary Materials

The following supporting information can be downloaded at: https://www.mdpi.com/article/10.3390/ijms252011140/s1.
