# Supplementary material for: Influence of Common Gene Variants on Lipid Levels and Risk of Coronary Heart Disease in Afro-Caribbeans
Source: Int J Mol Sci. 2024 Oct 17;25(20):11140. doi: 10.3390/ijms252011140 (PMC11508861; doi:10.3390/ijms252011140)
Supplement: Supplementary file 1 [file ijms-25-11140-s001.zip › Supplementary materials.pdf]

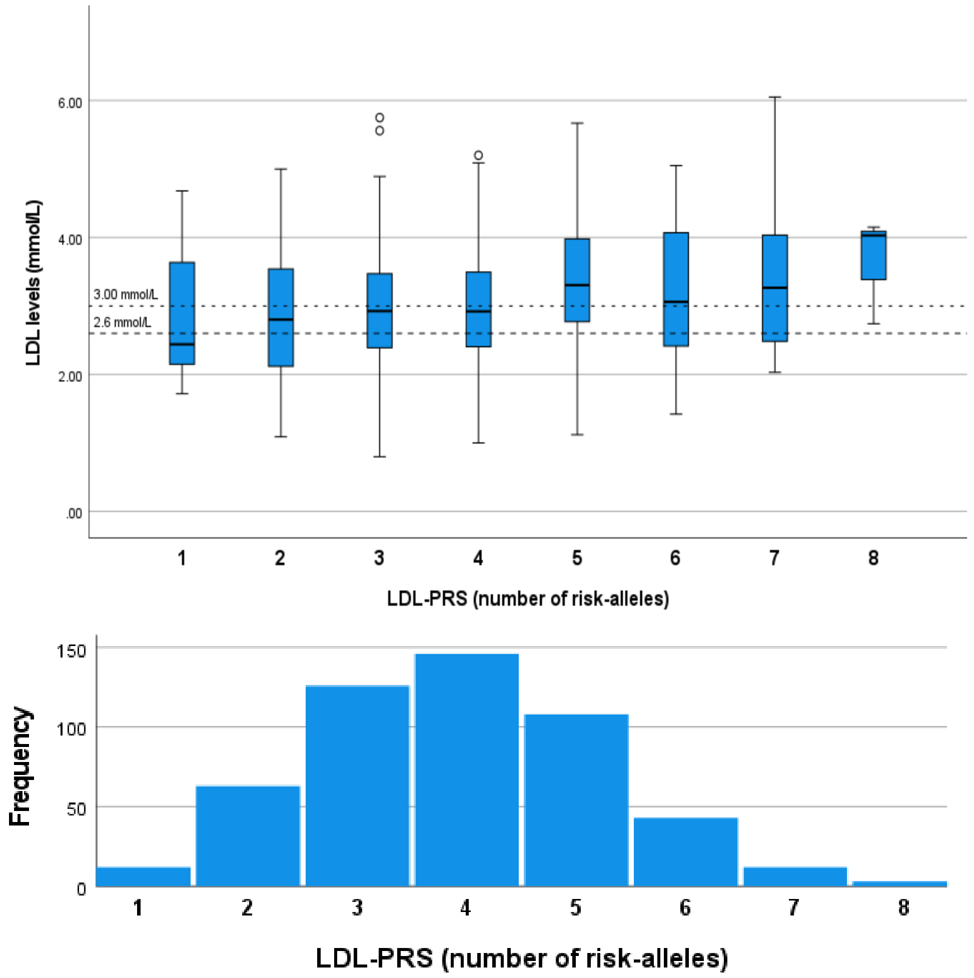

Figure S1: **frequency of individuals by LDL-PRS (risk allele count) and box plots of corresponding LDL levels**
Dotted lines indicate interventions thresholds according to ESC/EAS guidelines (Mach et al. 2020).
LDL: low density lipoprotein; PRS: polygenic risk score

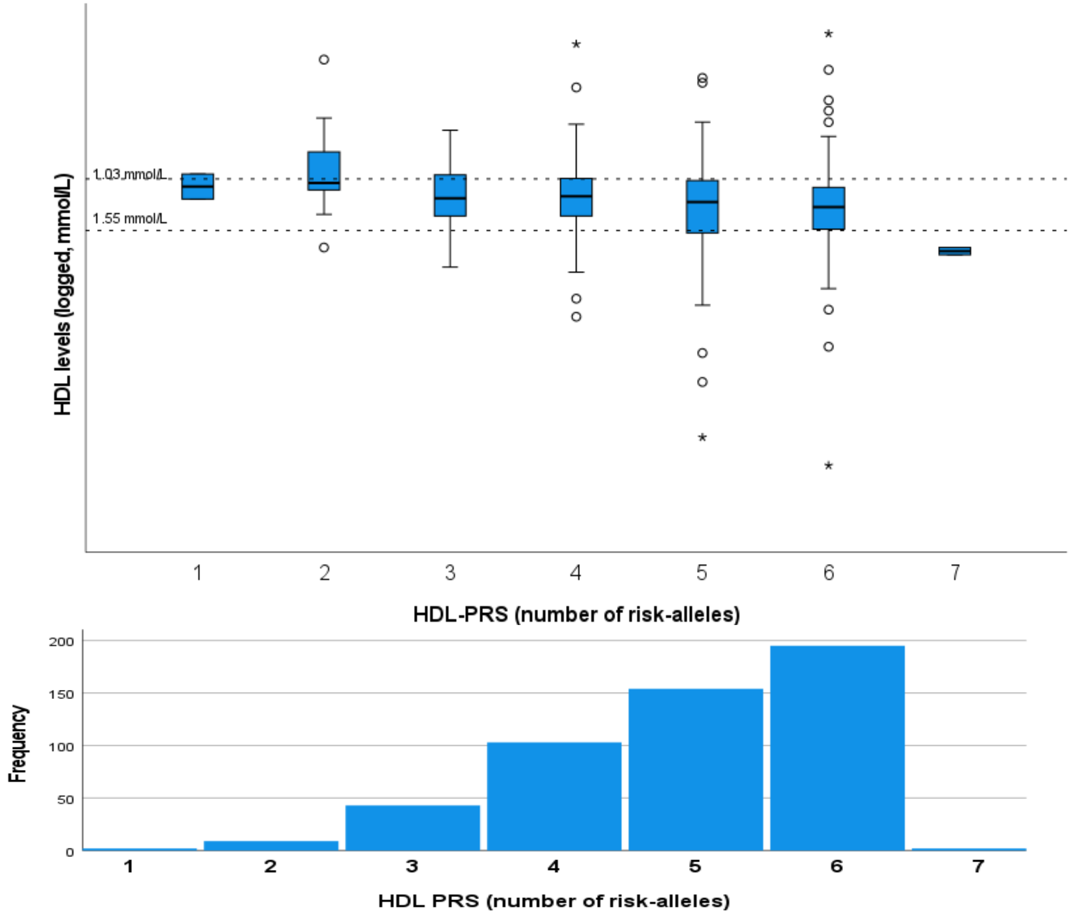

Figure S2: **frequency of individuals by HDL-PRS (risk allele count) and box plots of corresponding HDL levels**
HDL levels were log-transformed. Dotted lines indicate thresholds values associated with cardiovascular risk : HDL <1.03 mmol (40 mg/dL) is considered as a risk factor and HDL >1.55 mmol/L (60 mg/dL) is considered as a protective factor.
HDL: high density lipoprotein; PRS : polygenic risk score

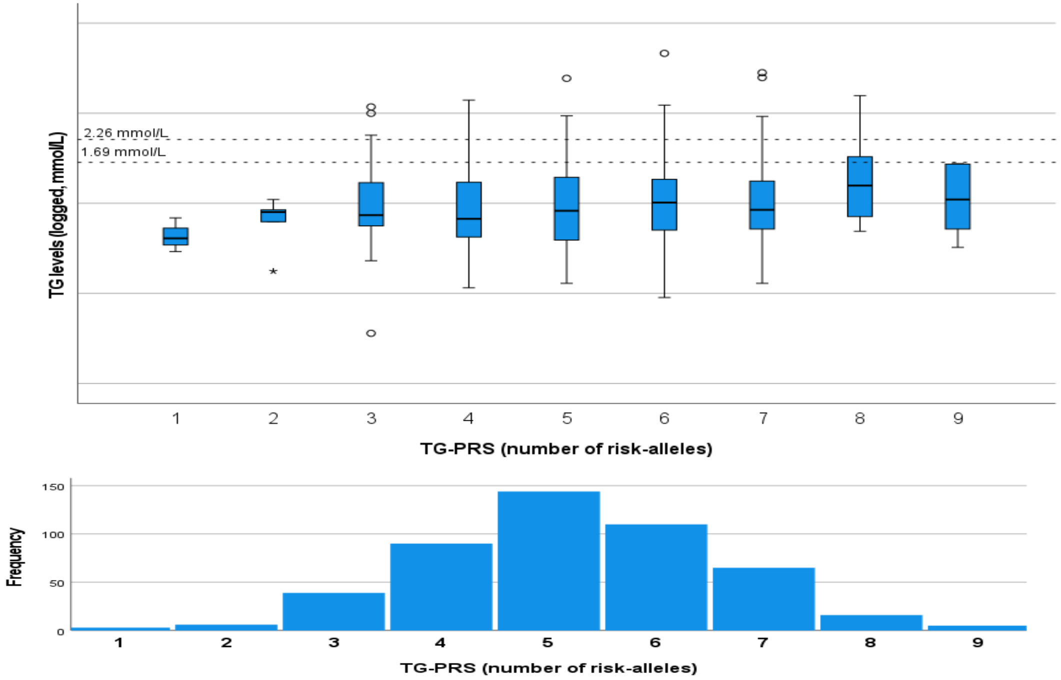

Figure S3: **frequency of individuals by TG-PRS (risk allele count) and box plots of corresponding TG levels.**
TG levels were log-transformed. Dotted lines indicate recognized cut point values : TG > 1.69 mmol/L (150 mg/dL) is considered as a risk factor for cardiovascular disease and the use of lipid lowering agent maybe considered if TG > 2.26 mmol/L (200mg/dL) in high-risk patients.
TG: triglycerides; PRS: polygenic risk score

Mach, F., C. Baigent, A. L. Catapano, K. C. Koskinas, M. Casula, L. Badimon, M. J. Chapman, G. G. De Backer, V. Delgado, B. A. Ference, I. M. Graham, A. Halliday, U. Landmesser, B. Mihaylova, T. R. Pedersen, G. Riccardi, D. J. Richter, M. S. Sabatine, M. R. Taskinen, L. Tokgozoglu, O. Wiklund, and E. S. C. Scientific Document Group. 2020. '2019 ESC/EAS Guidelines for the management of dyslipidaemias: lipid modification to reduce cardiovascular risk', *Eur Heart J*, 41: 111-88.
